# Lesional senescent CD4^+^ T cells mediate bystander cytolysis and contribute to the skin pathology of human cutaneous leishmaniasis

**DOI:** 10.3389/fimmu.2024.1475146

**Published:** 2024-10-21

**Authors:** Luciana Polaco Covre, Carlos Henrique Fantecelle, Renan Garcia de Moura, Paola Oliveira Lopes, Isabela Valim Sarmento, Celio Geraldo Freire-de-Lima, Debora Decote-Ricardo, Herbert Leonel de Matos Guedes, Alessandra Marcia da Fonsceca-Martins, Lucas Pedreira de Carvalho, Edgar Marcelino de Carvalho, David M. Mosser, Aloisio Falqueto, Arne N. Akbar, Daniel Claudio Oliveira Gomes

**Affiliations:** ^1^ Núcleo de Doenças Infecciosas, Universidade Federal do Espírito Santo, Vitória, Brazil; ^2^ Instituto de Biofísica Carlos Chagas Filho, Universidade Federal do Rio de Janeiro, Rio de Janeiro, Brazil; ^3^ Division of Medicine, University College London, London, United Kingdom; ^4^ Núcleo de Biotecnologia, Universidade Federal do Espírito Santo, Vitória, Brazil; ^5^ Departamento de Veterinária, Universidade Federal Rural do Rio de Janeiro, Rio de Janeiro, Brazil; ^6^ Instituto de Microbiologia Professor Paulo de Goes, Universidade Federal do Rio de Janeiro, Rio de Janeiro, Brazil; ^7^ Instituto Oswaldo Cruz, Fundação Oswaldo Cruz, Rio de Janeiro, Brazil; ^8^ Laboratório de Pesquisas Clínicas do Instituto Gonçalo Muniz, Fundação Oswaldo Cruz, Salvador, Brazil; ^9^ Department of Cell Biology and Molecular Genetics, University of Maryland, College Park, MD, United States; ^10^ Departamento de Medicina Social, Universidade Federal do Espírito Santo, Vitória, Brazil

**Keywords:** CD4-CTL, cutaneous leishmaniasis, *Leishmania braziliensis*, senescent cells, bystander cytotoxicity

## Abstract

Cytotoxic activity is a hallmark of the immunopathogenesis in human cutaneous leishmaniasis (CL). In this study, we identified accumulation of CD4^+^ granzyme B producing T cells with increased cytotoxic capacity in CL lesions. These cells showed enhanced expression of activating NK receptors (NKG2D and NKG2C), diminished expression of inhibitory NKG2A, along with the upregulation of the senescence marker CD57. Notably, CD4^+^ T cells freshly isolated from CL lesions demonstrated remarkable capacity to mediate NL-like bystander cytolysis. Phenotypic analyses revealed that lesional CD4^+^ T cells are mainly composed of late-differentiated effector (CD27-CD45RA-) and terminally differentiated (senescent) TEMRA (CD27-CD45RA+) subsets. Interestingly, the TEMRA CD4^+^ T cells exhibited higher expression of granzyme B and CD107a. Collectively, our results provide the first evidence that senescent cytotoxic CD4^+^ T cells may support the skin pathology of human cutaneous leishmaniasis and, together with our previous findings, support the notion that multiple subsets of cytotoxic senescent cells may be involved in inducing the skin lesions in these patients.

## 1 Introduction

In Leishmania infection, the immune response is critical in determining whether the outcome will be protective or pathological (1). Regarding the last, there is substantial evidence linking an overwhelming inflammatory response with the activation of the NLRP3 inflammasome and increased production of TNF-α and IL-1β (2–4). Additionally, the cytotoxic-driven pathology is observed in the skin lesions of patients (5–7), where comparative analyses between mucocutaneous leishmaniasis (MCL) and cutaneous leishmaniasis (CL) show an enrichment of CD8+ T cells with significant pro-inflammatory and cytotoxic activities and tissue destruction in MCL lesions compared to CL lesions (6, 8–10).

Both CD8^+^ and CD4^+^ T cells can be subdivided into four populations on the basis of their relative surface expression of CD27 and CD45RA molecules. This can define naive (CD45RA^+^CD27^+^), central memory T cells (TCM; CD45RA^−^CD27^+^), effector memory T cells (TEM; CD45RA^−^CD27^−^), and effector memory T cells that re-express CD45RA (TEMRA; CD45RA^+^CD27^−^). The latter contains the majority of senescent T cell populations and represent the final differentiated subset within the memory T cell compartment, which accumulates during ageing, persistent inflammatory disorders, and chronic infection (11). This subset exhibit features of senescence including the loss of proliferative capacity, DNA damage, reduced TCR function (12). Additionally, these senescent-like T cells acquire pronounced inflammatory and cytotoxic capacities and express NK cell receptors (NKRs) such as NKG2D and members of the KIR family (13). This enables them to mediate cytotoxic functions in an antigen-independent manner (13–15).

Although terminal differentiation is more commonly observed in the CD8^+^ T cell pool, CD4^+^ T cells can also reach an end-stage of differentiation in inflammatory or infectious contexts, where they express NK cell receptors (NKRs) and exhibit non-antigen-specific cytotoxic functions (16, 17). This has been associated with the pathology in autoimmune disorders, cancer (18), viral (19) and parasitic infections (20–22).

Recently, we demonstrated the accumulation of both CD4^+^ and CD8^+^ circulating senescent T cells with a high inflammatory profile during infection with *L. braziliensis* (23). These cells exhibited an increased propensity to migrate to the skin (23), where they demonstrated increased inflammatory and cytotoxic activity (24, 25). Specifically, the accumulation of lesional senescent CD8^+^ T cells positively correlated with the size of the lesion (24). Thus, while the role of this subset in mediating the immunopathogenesis of cutaneous leishmaniasis (CL) is evident, it remains unclear whether CD4^+^ cells acquire cytotoxic capacity and if they may also contribute to exacerbating the severity of skin lesions.

In this study, we demonstrate that the transcriptomic signatures of cytotoxic markers in the lesions of patients with cutaneous leishmaniasis correlate with CD4^+^ T cell signature genes. Our findings reveal an accumulation of CD4^+^ T cells exhibiting senescent features and a capacity to mediate bystander cytolysis through NK receptor activation, contributing to lesional immunopathology. Collectively, our results provide the first evidence that senescent cytotoxic CD4^+^ T cells may play a significant role in the skin pathology associated with human cutaneous leishmaniasis.

## 2 Materials and methods

### 2.1 Study subjects

Peripheral blood from 10 untreated patients with cutaneous leishmaniasis (CL) attended at the University Hospital (HUCAM) of Universidade Federal do Espírito Santo, Brazil, were investigated in this study. The diagnosis of CL was based on clinical and laboratory criteria and all patients in this study tested positive for the PCR/restriction fragment length polymorphism of *L. braziliensis* and reported no prior infections or treatments. The control group consisted of healthy age- and gender-matched individuals (HC) living in a non-endemic area without a history of leishmaniasis. All participants were seronegative for HIV, HBV and HCV infections and had no history of chemotherapy, radiotherapy or treatment with immunosuppressive medications within the last 6 months. The patient and control samples were obtained before the COVID-19 outbreak. Patients provided written informed consent, and study procedures were performed in accordance with the principles of the Declaration of Helsinki. This study was registered at HUCAM ethical committee reference number 735.274.

### 2.2 PBMC isolation and cell sorting

Peripheral blood was obtained using a Vacutainer blood collection system and EDTA-treated tubes. PBMCs were isolated by centrifuging whole blood through a Ficoll-Hypaque gradient (GE Healthcare), and cell viability was assessed using trypan blue dye exclusion. Cells were resuspended in RPMI medium and immediately processed according to the requirements of each experiment. Cell populations were obtained from fresh PBMCs using magnetic-activated cell sorting (MACS, Miltenyi Biotec) with a negative selection procedure, following the manufacturer’s protocol.

### 2.3 Biopsies collection and skin dissociation

Prior to therapy, a 6 mm skin punch biopsy was obtained from the border of cutaneous leishmaniasis lesions by a qualified clinician. Additionally, a 4 mm skin punch biopsy was collected from volunteers and used as an experimental control. The tissue samples were placed in a 15 mL Falcon tube containing 3 mL of 1X PBS, kept on ice, and immediately transferred to the laboratory for further processing. The biopsies were treated with collagenase for 4 hours, dissociated, and passed through a 70 μm cell strainer (BD Pharmingen). The cells were then washed by centrifugation and resuspended in RPMI medium. Cells were counted using a hemocytometer, and the cell suspension was adjusted as needed for further experiments.

### 2.4 Flow cytometric analysis

Multi-parameter flow cytometry was used for phenotypic and functional analyses using fresh PBMCs and fresh lesion-derived cells. For analysis of surface markers, staining was performed at 4°C for 30 min in the presence of saturating concentrations of a live/dead fixable Zombie NIR (BioLegend) and the following antibodies: anti-CD45 (HI30); anti-CD3 (UCHT1); anti-CD4 (RPA-T4); anti-CD8 (RPA-T8); anti-CD56 (HCD56); anti-CD107a; anti-CD27 (L128); anti-CD45RA (HI100) from BD Biosciences. Anti-NKG2D (149810); anti-NKG2C (134591); anti-NKG2A (131411) from R&D Systems. For intracellular analysis of cytotoxic granule expression, cells were fixed and permeabilized with the Fix & Perm^®^ Kit (Invitrogen, Life Technologies, UK), before incubation with granzyme B (GB11). Samples were acquired in a CytoFLEX LX flow cytometer (Beckman Coulter) and analysed using FlowJo software (TreeStar). ICRs gates were based on pooled fluorescence minus one control samples and applied identically across all samples. Gate strategy is described in the **Supplementary Figure S1**.

### 2.5 RNA-Seq analysis

The counts matrix used for transcriptomics analyses were obtained from a previous study at NCBI’s Gene Expression Omnibus (GEO) and the accession code GSE127831 [29]. All data was processed as described previously (26). Briefly, the dataset was comprised of transcript abundance of skin from healthy controls and lesions of patients with CL. DESeq2 (27) was used to determine differential expression, where genes with adjusted p-value less than 0.05 threshold were considered significant. The cytotoxicity signature scores were calculated using the GSVA package for the pre-selected genes using the “gsva” method. Violin plots and heatmaps were generated using ggplot2 and ComplexHeatmaps (28) respectively. Deconvolution of bulk RNA-Seq data was performed using the SCADEN (29) implementation in the R package omnideconv (30) and the Skin Single-Cell Atlas (31) as a reference. To reduce the size of the atlas dataset, each cell type was randomly subset using Scanpy (32) to preserve at most two thousand cells. The reduced dataset was used to build the reference matrix and infer cell population proportions.

### 2.6 Calcein-release cytotoxicity assay

The cytotoxic activity was assessed using the K562 cells (human erythroleukaemia cell line) as target. Briefly, 20.000 K562 cells were labelled with Calcein-AM (Sigma-Aldrich, St Louis, MO) at 10 μM for 1 h and plated in a 96-well flat-bottom plate for co-culture with NK^+^ or CD4^+^ cells in complete medium containing 500 IU/ml recombinant human interleukin-2 (rhIL-2) (Miltenyi Biotech). Effector and target cells were combined at a ratio of 40:1 in triplicate. After 4 hr of co-culture, fluorescence was measured in 75 μl of cell culture supernatant using a Spectramax Gemini spectrofluorimeter. Specific lysis was calculated as % killing = (test release–spontaneous release)/(max release–spontaneous release) x 100.

### 2.7 Multiplex immunofluorescence

Subjects diagnosed with cutaneous leishmaniasis (n = 4) were recruited from the Reference Center for Diagnosis and Treatment of c Leishmaniasis in Corte de Pedra - BA, Brazil. Written informed consent was obtained from all participants. Ethical approval for this study was granted by the Hospital Universitário Professor Edgard Santos’s Ethical Committee under the referential number 13926519.0.0000.5577. FPA- paraffin embedded histological sections were obtained from skin biopsies and prepared on poly-L-lysine coated glass slides. These FPA-paraffin embedded sections were dewaxed and rehydrated using xylene ethanol series for immunofluorescence. Permeabilization was performed using a PBS solution with 0.3% Triton X-100. Antigen retrieval was accomplished using Tris-EDTA (pH9) and Citrate (pH6) buffers under pressure conditions. Sections were stained with conjugated antibodies anti-CD4 (ab280849, Abcam), anti-CD8 (372906, BioLegend), anti-CD68 (ab277276, Abcam) and anti-GranzymeB (14-8822-82, Thermo Fisher) for 18 h at 4°C. Slides were mounted with Fluoroshield Mounting Medium containing DAPI followed by image acquisition using the Axioscan 7, Zeiss, Germany. Segmentation analysis was developed using the Highplex FL module from HALO Software (Indica Labs) where the CD4 T cell population was defined by CD4^+,^ CD8^-^ and CD68^-^ cells.

### 2.8 Statistics

GraphPad Prism (version 7) was used to perform statistical analysis. Data distribution was verified using the Shapiro-Wilk test. Statistical significance was evaluated using the paired Student t-test. Wilcoxon matched-pairs test was used for paired continuous nonparametric variables, while Friedman test or Kruskal-Wallis test was performed for comparing multiple groups of continuous nonparametric variables. Differences were considered significant when p was < 0.05. Gene signature scores statistical differences were calculated using Wilcoxon’s Rank Sum test in R. The correlation between the signature and the estimated cell type proportions was assessed with Spearman’s Rank Correlation test in R. Significance levels were represented as: *: p < 0.05; **: p < 0.01; ***: p < 0.001; ****: p < 0.0001.

## 3 Results

### 3.1 Lesional skin tissue demonstrates a pronounced cytotoxic signature that correlates with CD4^+^ T cells

**Figure 1:**
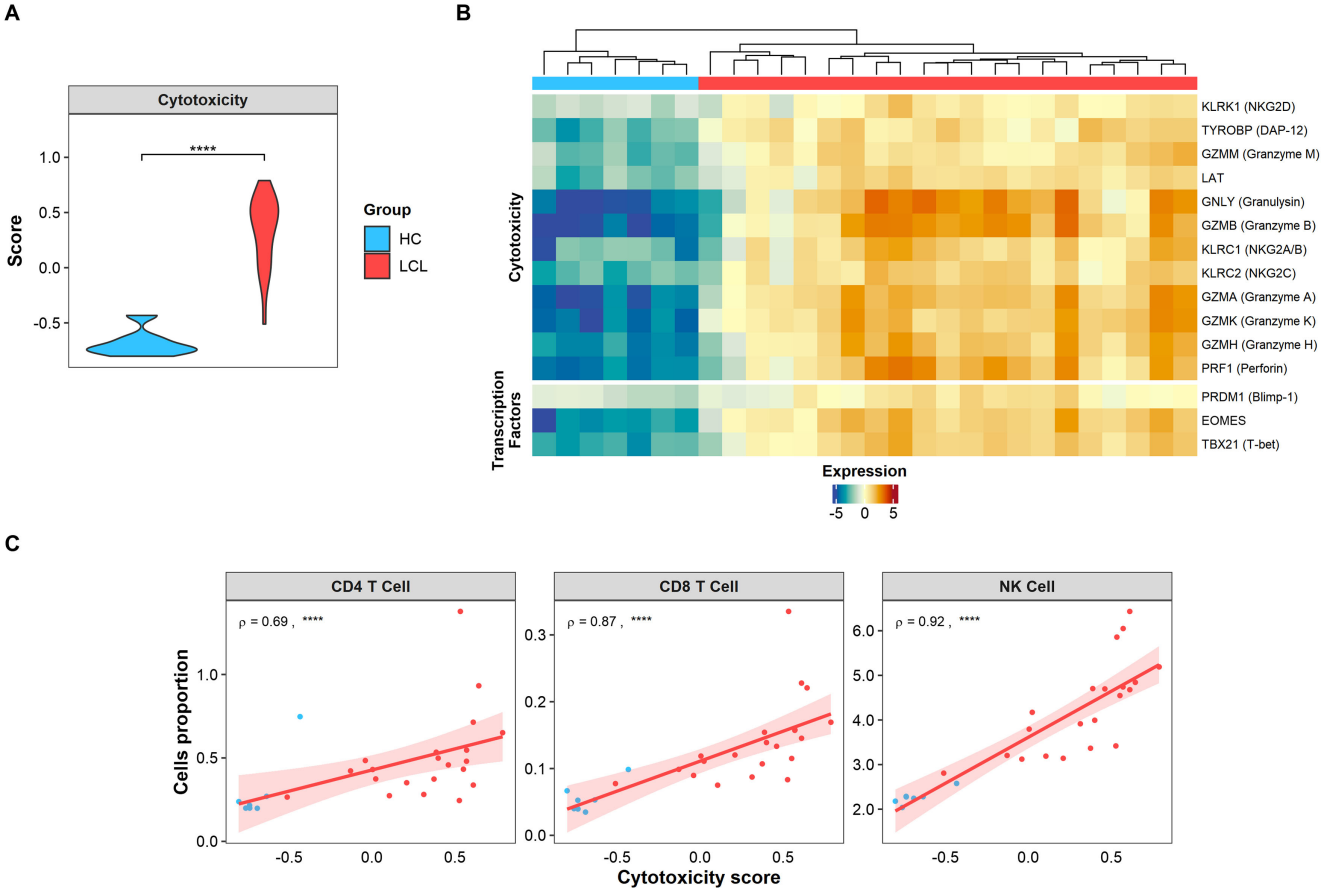
Cytotoxic signature is found in CL lesions and positively correlates with CD4 T cells. **(A)** Cytotoxic signature score expressed in skin biopsies from healthy controls (HC, light blue) and lesions from LCL patients (LCL, red). Gene signature scores statistical differences were calculated using Wilcoxon’s Rank Sum test. **(B)** The heatmap of cytotoxicity and activation genes differentially expressed in skin lesions represented as relative log2 normalized and variance stabilized (vst) values, where healthy controls (HC, light blue) and lesions from LCL patients (LCL, red) are compared. **(C)** Spearman’s correlation test between cytotoxic score and estimated cell (NK, CD4^+^ and CD8^+^) proportions obtained by cell deconvolution, where ρ represents Spearman’s Rho value. The p values were calculated using ANOVA test with Bonferroni correction **** *p* < 0.0001.

Cytotoxicity has been identified as one of the primary immunopathological signatures of CL lesions (9, 33). Supporting this, our lesional transcriptomic analysis using GSVA revealed an elevated score for this signature (**Figure 1A**), along with the overexpression of cytotoxic-associated genes compared to healthy skin (**Figure 1B**). Moreover, transcription factors associated cytotoxic activity (Blimp-1 and T-bet) and the terminal differentiation state of T cells (EOMES) were conspicuously expressed in the CL lesional environment, but not in healthy skin (**Figure 1B**). Interestingly, the cytotoxicity score positively correlated with the estimated cell proportion of conventional cytotoxic CD8+ and NK cells present in CL lesions (**Figure 1C**). Surprisingly, we also found a strong positive correlation between the cytotoxicity score and the CD4+ T cell signature, suggesting that this population may also be involved in lesional cytotoxic activity (**Figure 1C**).

**Figure 2:**
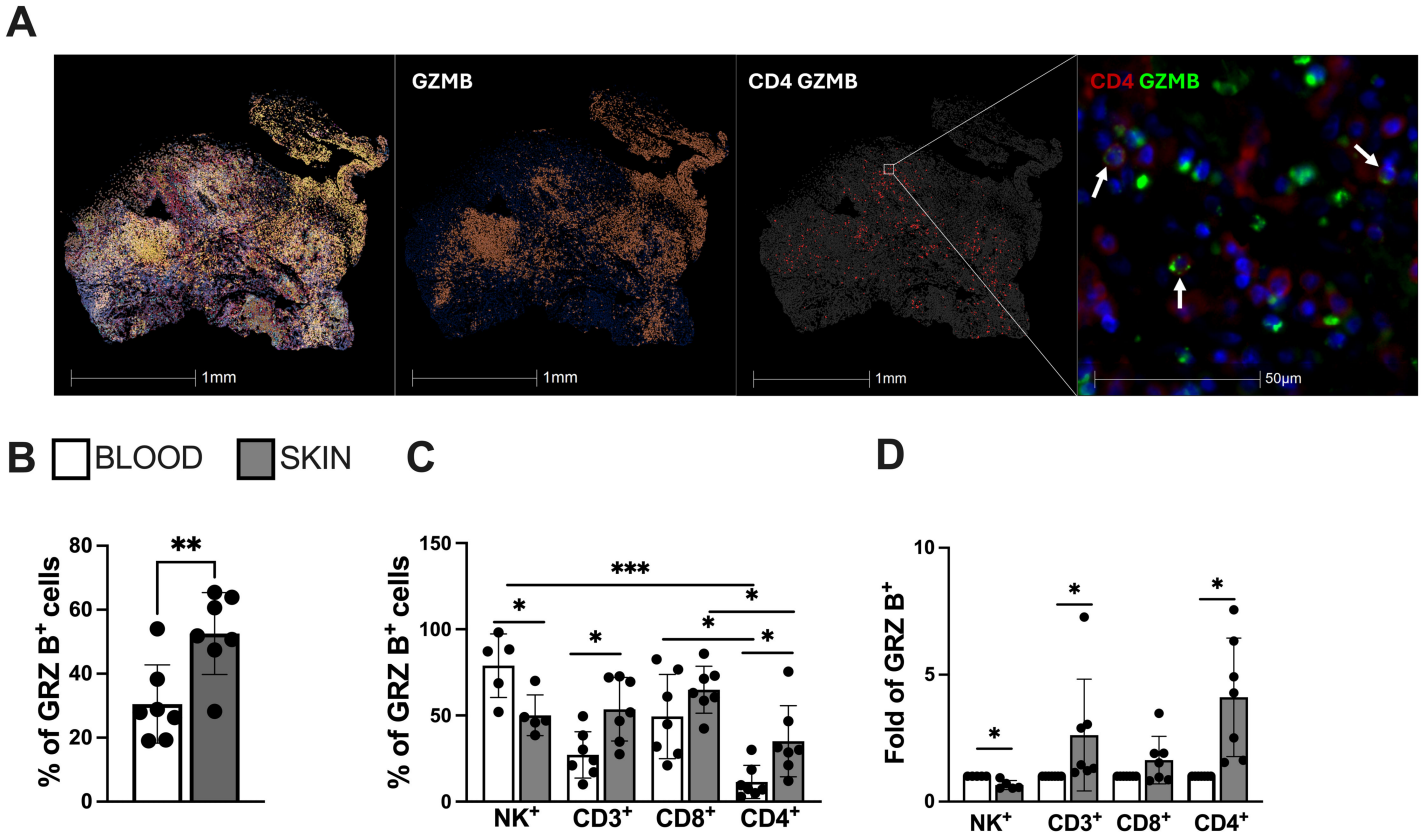
Lesional skin-infiltrating CD4+ T cells from CL-patients exhibit enhanced expression of cytotoxicity and natural killer (NK) markers. **(A)** Representative image of cellular infiltration, dispersion of granzyme B positive cells (red) and cytotoxic CD4^+^ T cells in CL lesions (green arrows indication). Staining was performed using immunofluorescence assay and analysed by Halo software. **(B)** Frequency of granzyme B and **(C)** cells expressing granzyme B in the blood and lesions of CL patients and **(D)** granzyme B differential expression (fold change) within circulating and lesional CD4^+^ and CD8^+^ T cells. The graphs show the mean ± SD. The p values were calculated using t test or ANOVA with Bonferroni correction (*p< 0,05, **p<0.01) ***p<0.001).

Histological analysis of the CL lesional skin demonstrated intense cellular infiltration with a conspicuous presence of CD4-GzB^+^ T cells (**Figure 2A**), further supported by flow cytometry analysis of the cellular suspension obtained by enzymatic digestion. This reveals an increased frequency of GzB^+^ cells compared to their blood counterparts (**Figure 2B**), found in the NK^+^, CD3^+^, CD8^+^, and CD4^+^ cell compartments (**Figure 2C**). Interestingly, while granzyme B-producing NK and CD8^+^ T cells were increased in both the blood and skin lesions of the same CL patients, CD4^+^ T cells producing granzyme B were only elevated in the skin of these individuals (**Figure 2D**).

### 3.2 Lesional CD4^+^-CTL cells display characteristics of senescence and mediate bystander cytotoxicity

**Figure 3:**
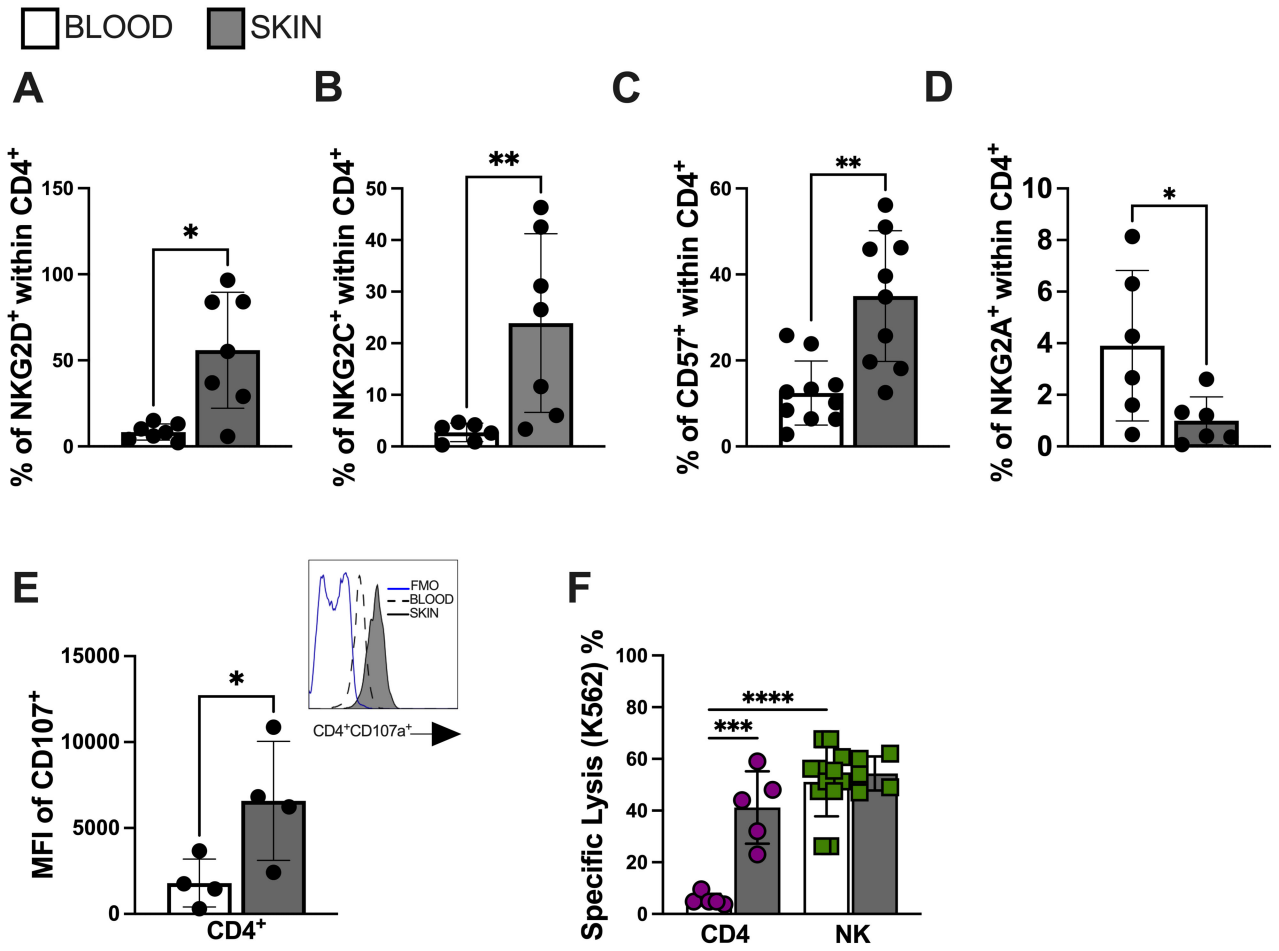
Lesional CD4 T cells mediate bystander cytotoxicity through NK receptors. Frequency of **(A)** NKG2D, **(B)** NKG2C, **(C)** CD57 and **(D)** NKG2A within lesional CD4+ T cell. **(E)** Lesional CD4^+^ T cell degranulation capacity accessed by CD107 mean fluorescence intensity (MFI); representative histogram of CD107a expression (blue line represents FMO; dashed line represents blood cells; solid line represents skin cells) and **(F)** bystander cytolysis capacity against K562 cell line. Purified CD4^+^ T cells (over 95% purity) from CL lesions were co-cultured with K562 target cells (effector: target ratio of 10:1) for 18 hr. The cytotoxic activity assessed by calcein-release lysis assay. The graphs show the mean ± SD. The p values were calculated using paired t test or ANOVA with Bonferroni correction (*p< 0,05, **p<0.01) ***p<0.001).

Senescent T cells acquire the expression of end-stage differentiation markers such as CD57 (34). Furthermore, they also acquire an NK-like phenotypic and functional profile that enables them to perform cytotoxic activity in a non-antigen-specific manner (13, 15). We next conducted a comparative analysis of CD4^+^ T cell populations from lesional and circulating compartments of CL patients. We found increased expression of activation receptors NKG2D (**Figure 3A**) and NKG2C (**Figure 3B**), as well as elevated expression of CD57 (**Figure 3C**) within lesional CD4^+^ T cells compared to their circulating counterparts. No significant difference was observed in the expression of the NKG2A inhibitory receptor (**Figure 3D**). Interestingly, lesional CD4^+^ T cells exhibited evidence of degranulation capacity (**Figure 3E**). Moreover, functional analysis revealed elevated NK-like cytotoxic activity against K562 target cells compared to the circulating CD4^+^ T cell populations from the same individuals (**Figure 3F**). These findings suggest that the lesional environment in CL is crucial in fostering the distinctive bystander cytolysis and senescence features observed in the CD4^+^ T cell population that may contribute to non-specific pathology through NK-like killing activity.

### 3.3 Lesional CD4-CTLs are predominantly found within the senescent subset

**Figure 4:**
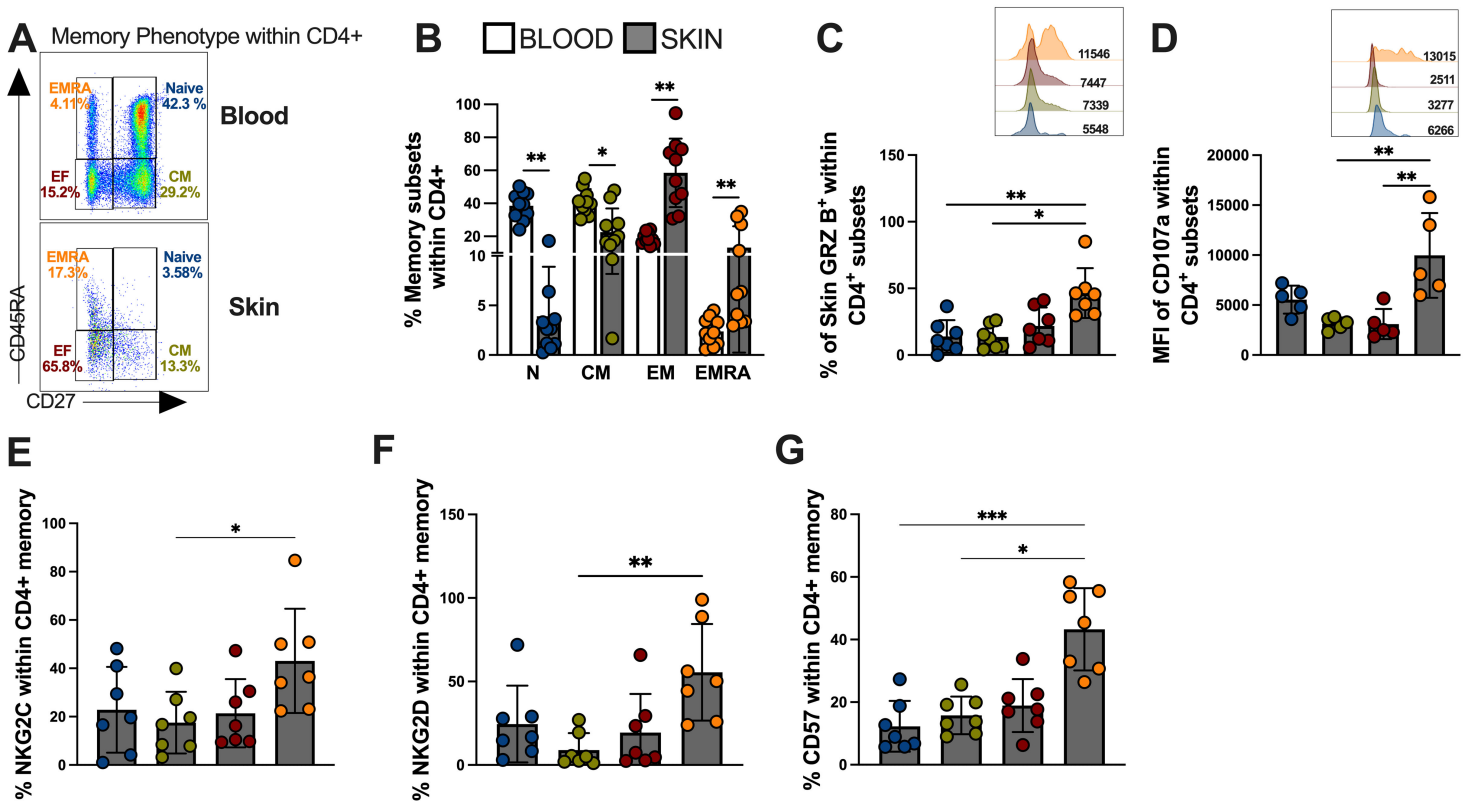
Skin lesion-derived CD4^+^ CTL T cells are predominantly found in the senescent-EMRA memory subset and demonstrate significant cytotoxic capacity. **(A)** Representative FACS plot and frequencies of memory T cell subsets defined by CD45RA/CD27 expression as: Naive (CD27 + 45RA+; Blue color), Central Memory (CD27+CD45RA-;Green color), Effector Memory (CD27-CD45RA; Red color), and Terminal Effector Memory EMRA (CD27-CD45RA+; Orange color) within CD4^+^ T cells and **(B)** cumulative data of memory subsets frequencies from blood and skin of CL patients. **(C)** Frequency (%) and representative histogram of granzyme B mean fluorescence intensity (MFI). **(D)** Cumulative data and representative histogram of Mean Fluorescence Intensity (MFI) of the expression of CD107a. Cumulative data of **(E)** NKG2C, **(F)** NKG2D and **(G)** CD57 expression within memory CD4^+^ T cells subsets. The p values were calculated using ANOVA test with Bonferroni correction (*p< 0,05, **p<0.01, ***p<0.001, **** *p* < 0.0001).

T cells can be subdivided into four populations based on their relative surface expression of CD27 and CD45RA molecules (35). This can define naive (CD45RA^+^CD27^+^), central memory (CM; CD45RA^−^CD27^+^), effector memory (EM; CD45RA^−^CD27^−^), and effector memory T cells that re-express CD45RA (EMRA; CD45RA^+^CD27^−^) that contain the majority of senescent T cells and represents the end-differentiated memory subset (35). Next, we investigated the memory heterogeneity of lesional CD4-CTLs. Interestingly, the differentiated CD4^+^ T cell populations, represented by effector memory (EM) and terminal effector memory (EMRA), were more frequent in the skin compared to the blood of the same individuals (**Figures 4A, B**). Moreover, EM and EMRA CD4^+^ T cell subsets exhibited increased granzyme production compared to naive and central memory (CM) subsets indicating greater cytotoxic capacity (**Figure 4C**). The observation of higher CD107 (**Figure 4D**), NKG2C (**Figure 4E**) and NKG2D (**Figure 4F**) expressions in EMRA populations confirmed this and further indicated that these cells were likely to have mediated cytotoxic degranulation. Furthermore, the EMRA subset showed pronounced expression of CD57 (**Figure 4G**), supporting the idea that cytotoxic activity within the CD4^+^ T pool is mediated by highly differentiated/senescent populations.

## 4 Discussion

It is well established that CD4^+^ T cells play a pivotal role in the protective response during human cutaneous leishmaniasis caused by *L. brasiliensis*. This is achieved by activating IFN-γ-dependent macrophage microbicidal mechanisms and enhancing the adaptive immune response for protection (36). Here, we demonstrate the association of CD4^+^ T cells with cytotoxic capacity in CL lesion environment, potentially implicating their contribution to the pathophysiology of cutaneous leishmaniasis.

The pathogenic impact of cytotoxicity is observed in the skin lesions of patients with cutaneous leishmaniasis, where cytotoxicity-associated genes are more upregulated compared to pro-inflammatory genes (37). The increased production of granzyme B and CD107a at the lesional site is positively correlated with tissue damage and the size of lesions observed in patients (38, 39). Furthermore, the accumulation of granzyme-producing CD8^+^ T cells has been associated with the lesional severity (24, 39). Interestingly, we found an unexpected accumulation of CD4^+^ T cells with cytotoxic capacity in the lesional environment. Although this population was proportionally smaller than CD8^+^ T or NK cells in the lesions, they demonstrated an equal capacity to mediate cytotoxicity compared to other cytotoxic subsets, and a superior capacity to their circulating counterparts. This raises the hypothesis that the tissue environment may significantly influence the acquisition of cytotoxic capacity within diverse T-cell compartments. An example is the impact of lesional hypoxia (40) on the targeting and acquisition of cytotoxic capacity by CD8+ T cells. This could also extend to CD4^+^ T cells and warrants further investigation.

Chronic antigenic exposure or persistent inflammatory conditions have been associated with the acquisition of cytotoxic characteristics by CD4^+^ T cells, which are implicated with pathology in various human diseases (41–43). These highly cytotoxic CD4^+^ T cells express classical markers of T cell (44–46). Indeed, previous studies by our group have shown both lesional and circulating accumulation of senescent CD4^+^ and CD8^+^ T cells correlate significantly with the tissue damage observed in patients (23, 24). Conversely, healthy volunteers present a very low frequencies of senescent T cells, which were not associated with cytotoxic or inflammatory activity (23). These observations align with other studies linking chronic infectious processes to the clinical severity caused by senescent cells. In this context, senescent-like CD4^+^-CTLs that are cross-reactive to different dengue serotypes have been identified in patients with severe dengue fever (47). These cells exhibit an enhanced capacity to mediate the killing of target cells through Fas/FasL recognition or perforin release (48, 49). Similar findings have been reported in hepatitis virus infections, where both senescent-like CD4^+^ T cells and CD8^+^ T cells expressing perforin are significantly elevated in patients compared to healthy controls (48). These cells target liver hepatocytes, contributing to disease pathology (50). Interestingly, CD4 cells with cytotoxic activity have been identified in several skin diseases, such as diabetic ulcers, dermatitis, and psoriasis, suggesting that they may mediate pathogenesis through mechanisms similar to those described in this work, which warrants further investigation.

Another interesting observation is that chronic infections direct T cells towards senescence. Under these conditions, senescent T cells, not only acquire an increase in functional capacity (as previously described) but also exhibit a profound NK cell signature (51). This signature is associated with adapter molecules, activation of intracellular pathways, and the expression of activation/inhibition ligands, enabling them to mediate NK cell activity and bystander cytolysis in an antigen-independent manner (51–53). This phenomenon is particularly intriguing in the context of human and murine infections by *Leishmania brasiliensis*, where the role of CD8^+^ T cells in mediating pathogenesis through this mechanism has been previously demonstrated (54, 55). In our experiments, we extended these findings by demonstrating that CD4^+^ T cells acquire characteristics that mediate tissue damage similar to that observed in the CD8^+^ and NK cell compartments.

**Figure 5:**
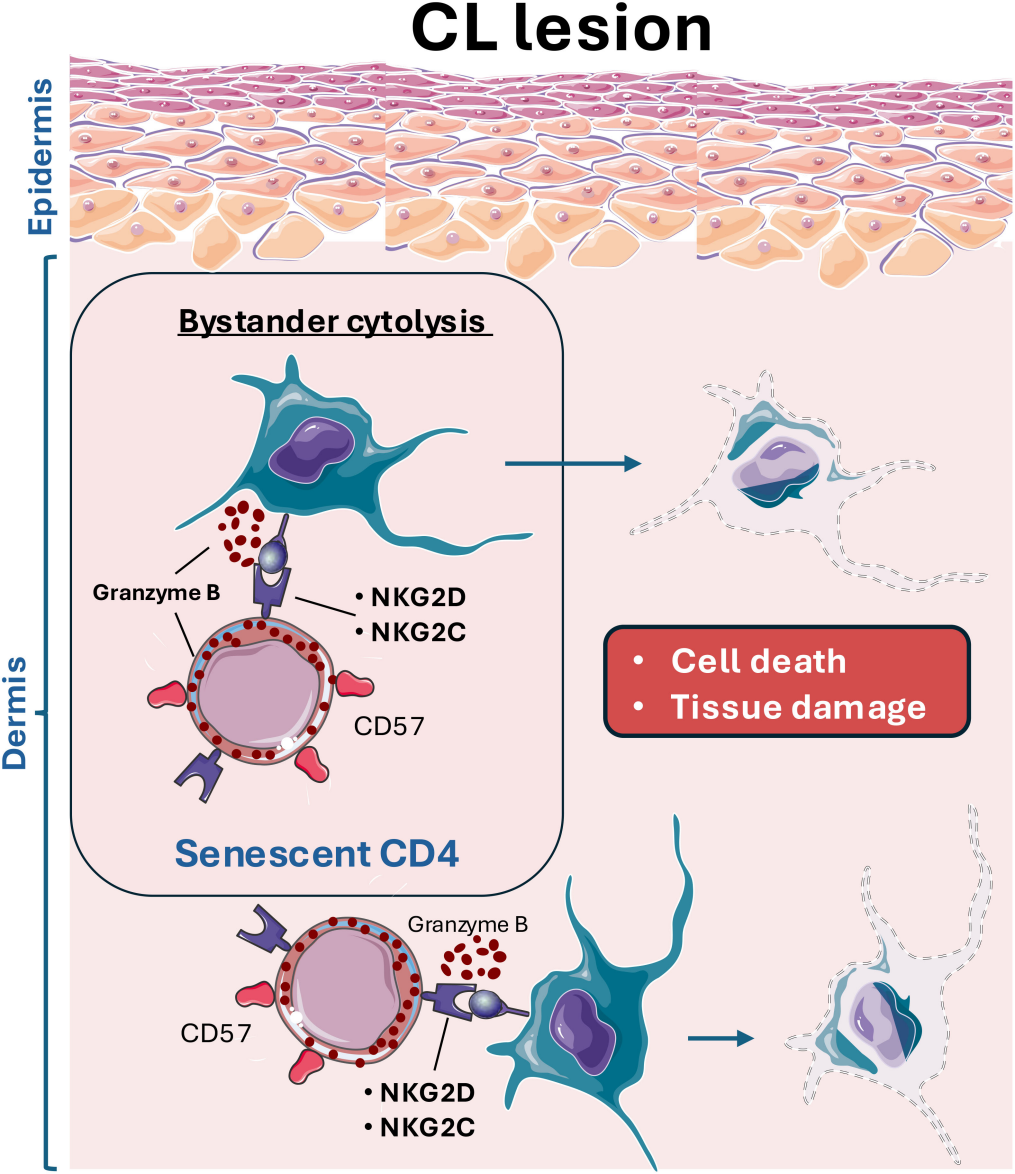
Leishmania infection leads to the infiltration of cytotoxic CD4 T cells (CD4-CTL) with senescent features, which aberrantly target and kill stromal and immune cells, contributing to the excessive tissue damage observed in cutaneous leishmaniasis lesions. These CD4-CTLs, expressing various NK receptors (NKRs), including NKG2D and NKG2C, interact with resident skin cells. This interaction results in bystander activation and skin cells killing, exacerbating off-target tissue pathology.

Overall, this study provides the first evidence that senescent cytotoxic CD4+ T cells contribute to mediating skin pathology in human cutaneous leishmaniasis through bystander cytolysis via NK receptors. The interaction of CD4-CTLs expressing NKG2C and NKG2D with other immune and stromal cells expressing their ligands could lead to cell death and, consequently, nonspecific tissue damage (**Figure 5**). Further studies investigating the role of CD4+ T cells in the immunopathogenesis of this disease could facilitate the development of novel therapeutic strategies or identify these cells as potential biomarkers for disease severity.

## Data Availability

The datasets presented in this study can be found in online repositories. The names of the repository/repositories and accession number(s) can be found in the article/**Supplementary Material**.

## Supplementary material

Supplementary Figure 1 Representative gate strategy for circulating and skin- CD4^+^ T cells, CD8^+^ T cells and NK cells.
