# Supplementary figures and images for: Lesional senescent CD4+ T cells mediate bystander cytolysis and contribute to the skin pathology of human cutaneous leishmaniasis
Source: Front Immunol. 2024 Oct 21;15:1475146. doi: 10.3389/fimmu.2024.1475146 (PMC11532160; doi:10.3389/fimmu.2024.1475146)

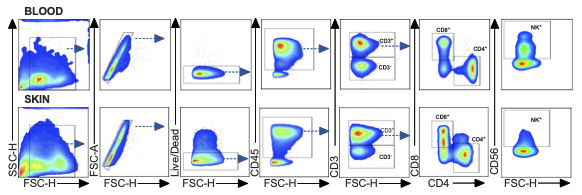

Supplement: Supplementary Figure 1 — Representative gate strategy for circulating and skin- CD4+ T cells, CD8+ T cells and NK cells. [file Image1.tiff]
